# Ludger Schuknecht, Public spending and the role of the state: history, performance, risk and remedies: Cambridge University Press, Cambridge, MA, 2020. xix + 284 pp, USD29.99 (paperback).

**DOI:** 10.1007/s11127-021-00898-7

**Published:** 2021-03-21

**Authors:** Niklas Potrafke

**Affiliations:** 1 Ifo Center for Public Finance and Political Economy, Ifo Institute, Poschingerstr. 5, 81679 Munich, Germany; 2 Department of Economics, University of Munich, Munich, Germany

Public expenditure has increased in many industrialized countries for decades. The COVID-19 crisis contributed to further increases in the size of government. Should we care? Yes. Ludger Schuknecht’s new book *Public Spending and the Role of the State*” inspires new thinking about the increasing size of government and what can be done. Schuknecht is very well prepared to elaborate on this topic. He is a brilliant scholar of Public Choice, having studied as an undergrad in the late 1980s with James Buchanan at George Mason University, and completing his Ph.D. with Henry Ursprung in Konstanz. Schuknecht was the Chief Economist of the German Ministry of Finance during the 2011–2018 period. Two decades ago, Ludger Schuknecht and Vito Tanzi published their book *Public Spending in the Twentieth Century*. Schuknecht’s new book continues this research by studying the determinants and consequences of public spending and the size of government.

Public Choice scholars will like this book. First, Schuknecht describes the increasing size of government in a vivid manner. He uses many descriptive statistics and chooses interesting individual examples. Second, his analysis is based on economic theory and he effectively links theory to descriptive evidence. Third, Schuknecht does not hesitate to advocate for a smaller size of government. He begins the individual chapters with quotes such as Milton Friedman’s “Government should be a referee, not an active player [in the economy]” and Angela Merkel’s “Europe needs to be competitive and we also need to be competitive if we wish to remain an interesting partner.” Schuknecht summarizes his basic argument as follows—“in order to continue the success story and maintain the trust of our citizens in a fast-changing world, we need to spend public money wisely. Governments should focus on their core tasks, be lean, efficient and financially sustainable, reform where needed and prevent undue risk. This requires sound rules and institutions for both government and the economy!” (p. 3).

To determine how governments should operate, Schuknecht discusses previous stories of success and cases that were less successful. The size of government increased drastically over the 1960–1980 period; it decreased in the 1980s and 1990s. Schuknecht differentiates “ambitious reformers,” those that decreased primary expenditure by at least 5% of GDP (Belgium, Ireland, the Netherlands, New Zealand already started in the 1980s—they were “early”), from “non-reformers,” those that did not decrease primary expenditure at all (Greece, Japan, Portugal), and from “timid-reformers,” those countries in between the ambitions reformers and non-reformers. Some of the ambitious reformers decreased expenditure and also implemented other (structural) reforms, such as introducing fiscal rules. In the ambitious reform countries, budget deficits decreased from 7% of GDP in the mid-1980s, to 1.5% of GDP in the mid-1990s (Chapter 5). These countries enjoyed economic growth and “the per capita income of the poor had increased by 43%, 9% points more than the average. The positive growth and employment effect on income of the poor seems to have dominated any worsening effect, for example from reductions in transfers” (p. 119). The descriptive statistics corroborate econometric evidence showing that budget consolidation was more successful by cutting expenditure than by raising revenues (e.g. Alesina et al. 2019).

Public expenditure and public debt increased drastically during the crisis beginning in 2007. The third wave of governmental reforms set in. Schuknecht investigates in detail the five “boom-bust” countries—Greece, Ireland, Portugal, Spain and the United Kingdom—as compared to the three big continental European countries—France, Germany and Italy. Public expenditure needed to be decreased in the 2010s. Interestingly, social expenditure was hardly cut within this period. By contrast, non-social expenditure decreased by almost 7% in the boom-bust countries. Decreasing public expenditure also helped to drastically reduce deficits and public debt. The boom-bust countries fared well. Greece may have been an exception, Schuknecht acknowledges, but “perhaps too little time has passed to show success” (p. 131). The reforms and strategies to consolidate budgets are certainly interesting against the background of the need to consolidate budgets after the COVID-19 crisis.

Schuknecht proposes specific numbers for the optimal size of governments—public expenditure should be 30–35% of GDP, maybe 40 when governments do well in fulfilling their core tasks, Australia and Switzerland being good examples. Ireland and Singapore are countries that do well, as Schuknecht describes, with a smaller sized government. And the Nordic countries do also well with a larger sized government. Schuknecht arrives at these numbers and assessments by using correlations between size of government and efficiency and performance indicators. The author is well aware of employing a “pragmatic” approach to arrive at these numbers (“these numbers should be seen as guidance rather than as ‘truth’” (p. 134)) and the assessment is quite similar to the one of Tanzi and Schuknecht (2000). It is conceivable that many scholars would hesitate to arrive at such clear-cut numbers based on the methods Schuknecht employs. In any event, Schuknecht should deserve credit for attempting to propose numbers on the optimal size of government.

Chapter 7 “Social Expenditure and the Risk of ‘Social Dominance’” is one of the most important chapters of the book. The chapter is based on the paper by Schuknecht and Zemanek (2021) in *Public Choice*. It is introduced by the Pope Francis quote, “Unrestrained liberalism only makes the strong stronger and the weak weaker and excludes the most excluded,” and Margaret Thatcher’s quote, “The problem with socialism is that you eventually run out of other people’s money.” Schuknecht shows the extent to which governments have put emphasis on social spending and describes the “social dominance” as one of three fiscal risks for the future. Governments in industrialized countries face the risk of being fiscally over-committed.

The benefits of public insurance are discussed. Schuknecht elaborates on risk sharing, adverse selection and moral hazard in relation to Public Choice theories. “The provision of insurance may follow political motives, rewarding groups of voters or special interests. Politicians can provide ‘favours’ by not pricing risks properly and by not monitoring or penalizing risk-increasing behavior on the part of the insured…The result can be a classic ‘common pool’ problem of too much and too inefficient public insurance” (p. 160). Social expenditure in established OECD countries increased from, on average, 9% of GDP in 1960, to 24% of GDP in 2016. Because of an aging population, projections suggest that social expenditure, especially those related to pensions and health care, will increase further in the future. Social expenditure crowds out money available for the other core tasks of government. “Looking forward, record debt, continuing financial risk and the growing fiscal burdens of population aging point to the next round of crisis if nothing is done” (p. 161). What needs to be done, according to Schuknecht, is placing more emphasis on public good provision rather than on transfers.

Schuknecht also presents results from the sustainability report of the German Ministry of Finance in 2016. Germany will not have a sustainability problem under favorable assumptions, but it clearly will under unfavorable assumptions. There is a new sustainability report of the German Ministry of Finance published in 2020. To be sure, Schuknecht does not refer to the new report in his book, but the results are telling—the sustainability gap in 2020 became larger as compared to the previous report in 2016.

In the final chapter, Schuknecht discusses the importance of rules and institutions. “Rules and institutions are the ‘red thread’ that can deal with challenges identified in this book” (p. 237). The author advocates fiscal rules to keep budgets balanced, to decrease risks in social insurance and financial systems, and to make sure that politicians focus on the governments’ core tasks. I could not agree more. In emphasizing the importance of rules and institutions, Schuknecht makes clear the importance of constitutional political economy for limiting undesirable government activity.
